# Nursing Progress and Its Developments: X. Local Centres for Examinations

**Published:** 1918-03-23

**Authors:** 


### THE MATRONS' AND SISTERS' DEPARTMENT.

### NURSING PROGRESS AND ITS DEVELOPMENTS.

### X.—Local Centres for Examinations.

THE institution of what is known as the "One Portal Examination" is one of the fundamental purposes of the College of Nursing. There are not wanting plausible advocates of the admirable nurses who it is claimed are valuable in the ward and sickroom, yet could never conceivably have passed any examination worthy of the name. But with every desire to be fair to a passing generation, the College is bound to build for the future. Nursing is now so highly technical in many of its developments that women who cannot master sufficient of its elements to express in plain words the things they will be called on to perform must be considered to have mistaken their vocation. There must be set, it is now almost universally agreed, an irreducible standard of nursing lore below which no woman must be stamped with the hall-mark of trained nurse. There must be one gate, however broad and accessible, through which candidates for every single branch of nursing must pass on their way to the ordinary routine or the highly specialised departments of the profession.

This principle having been clearly enunciated by the leading exponents of the College policy, there arose in some quarters a misapprehension about the meaning of the words "One Portal Examination." It has been taken to mean that every probationer on completing her course will be compelled to travel up from the part of the kingdom in which her training-school lies, to take lodgings in London and undergo the ordeal of two or three successive days of examination at headquarters. Such an enactment would enormously increase the expense of registration for each individual. Moreover it would seriously handicap the candidates from a distance, for the excitement and worry provoked by a short visit to the metropolis is the worst possible preparation for the examination room. The establishment of provincial examination centres is an essential feature in the local development of the College, and ought to be kept in mind from the beginning by those who are doing pioneer work in this direction.

It need not be assumed that every local centre which is formed will, in the end, develop into an examining centre. To act on this assumption would unduly restrict the activities of local representatives. Local centres, each with a strong nucleus of College members, may exist within a few miles of each other, yet find it more convenient to meet separately for discussions, professional meetings, and recreation. The examining centres will ultimately, we judge, be fixed by the council with a view to linking up different parts of the country round strong provincial centres, in such a way that every nurse who desires to be examined may find herself within a railway journey of, say, an hour or two from the examination room. The English way is not to sketch in advance grandiose schemes on paper, and then force everybody into conformity. It is rather to let the provinces organise themselves. Then when the work of local organisation is well in hand, when in many parts of the country lively and compact groups of nurses have coagulated, so to speak, under the ægis of the College, when the battle has been won and State registration is an accomplished fact, it will be the part of the Council to pick out, in various divisions of the Kingdom, central examining centres with which all other centres within a convenient radius can affiliate.

Thus the broad lines on which the One Portal Examination is likely to be brought within easy reach of every training school from north to south, from east to west, can be apprehended. Those who are working to lay the foundations of the College deep and strong in the provinces may be building to higher ends than they can immediately foresee.

